# Prognostic utility of systemic immune-inflammation markers in locally advanced cervical cancer undergoing radical radiotherapy

**DOI:** 10.1093/oncolo/oyag139

**Published:** 2026-04-21

**Authors:** Yue Li, Jing Han, Shuyue Xiao, Shanliang Zhong, Jianyao Liu, Huixin Li, Xinyi Xie, Zhen Gong, Hanzi Xu

**Affiliations:** Department of Radiation Oncology, The Affiliated Cancer Hospital of Nanjing Medical University & Jiangsu Cancer Hospital & Jiangsu Institute of Cancer Research, Nanjing 210009, China; Jiangsu Cancer Centre, The Affiliated Cancer Hospital of Nanjing Medical University & Jiangsu Cancer Hospital & Jiangsu Institute of Cancer Research, Nanjing 210009, China; Department of Gynecology, Women’s Hospital of Nanjing Medical University & Nanjing Women and Children’s Healthcare Hospital, Nanjing 210004, China; Center of Clinical Laboratory Science, The Affiliated Cancer Hospital of Nanjing Medical University & Jiangsu Cancer Hospital & Jiangsu Institute of Cancer Research, Nanjing 210009, China; Department of Radiation Oncology, The Affiliated Cancer Hospital of Nanjing Medical University & Jiangsu Cancer Hospital & Jiangsu Institute of Cancer Research, Nanjing 210009, China; Department of Gynecology, Women’s Hospital of Nanjing Medical University & Nanjing Women and Children’s Healthcare Hospital, Nanjing 210004, China; Department of Gynecology, Women’s Hospital of Nanjing Medical University & Nanjing Women and Children’s Healthcare Hospital, Nanjing 210004, China; Department of Gynecology, Women’s Hospital of Nanjing Medical University & Nanjing Women and Children’s Healthcare Hospital, Nanjing 210004, China; Department of Radiation Oncology, The Affiliated Cancer Hospital of Nanjing Medical University & Jiangsu Cancer Hospital & Jiangsu Institute of Cancer Research, Nanjing 210009, China

**Keywords:** cervical cancer, prognosis, systemic immune-inflammation markers, predictive model, nomogram, machine learning

## Abstract

**Purpose:**

To improve survival prediction in locally advanced cervical cancer (LACC) post-radiotherapy, we developed and compared a nomogram and machine learning (ML) models using immune-inflammatory markers.

**Material and Methods:**

Clinical data from patients with LACC who received radical radiotherapy at our department between 2016 and 2019 were retrospectively analyzed. Key variables were selected using Spearman correlation and Boruta analysis. A Cox regression-based nomogram was developed and validated. ML models were also used to predict survival, with performance evaluated by accuracy, the area under the receiver operating characteristic curve (AUC), precision, recall, and the F1-score. The performance of the optimal ML model and the nomogram was compared using time-dependent predictive accuracy.

**Results:**

A cohort of 300 patients with LACC was randomly allocated to training and validation cohorts at a 7:3 ratio. The nomogram and ML models incorporated immune-inflammatory markers and clinical variables. The nomogram demonstrated strong discriminative ability, calibration, and clinical utility, as evidenced by AUC, calibration plots, and decision curve analysis. Among ML models, logistic regression achieved superior performance (accuracy = 0.933, recall = 1.000, F1-score = 0.938). Time-dependent accuracy analysis revealed dynamic predictive performance over 12-60 months. The nomogram’s predictive accuracy improved over time, reaching 0.611 at 53-59 months, while logistic regression peaked earlier (0.911 at 12-24 months) and consistently outperformed the nomogram.

**Conclusion:**

Both nomogram and ML models provide valuable prognostic insights for LACC. The logistic regression ML model showed superior predictive accuracy compared to the nomogram during 1-5 years of follow-up.

Implications for Practice This study validated a prognostic model integrating immune-inflammatory markers with FIGO staging for survival prediction in locally advanced cervical cancer after radiotherapy. The nomogram demonstrated strong discrimination and accuracy for clinical use. Time-dependent analysis showed logistic regression ML model outperformed the nomogram at all intervals (12-60 months), suggesting its potential as a more precise tool for prognostic assessment and decision support.

## Introduction

Cervical cancer (CC) is the fourth most common malignancy and fourth leading cause of cancer mortality in women worldwide.^1–2^ Based on the 2018 International Federation of Gynecology and Obstetrics (FIGO) staging system, locally advanced cervical cancer (LACC) encompasses stages IIB to IVA.^3^ Globally, LACC constitutes approximately 37% of all CC cases, with the proportion reaching even higher levels in Asian countries.^4^ Despite undergoing standard concurrent chemoradiotherapy (CCRT), approximately 40% of LACC patients develop recurrence, with a post-relapse 5-year survival rate below 5%.^5^ This underscores the critical need for precise prognostic tools to optimize LACC management.

The FIGO staging system remains the cornerstone of clinical prognosis evaluation, yet it has significant limitations. Its complexity, heavy reliance on postoperative pathology, and inability to account for prognostic heterogeneity within the same stage hinder its accuracy—particularly in LACC, where radical radiotherapy precludes surgical pathological assessment. Key prognostic factors, including tumor dimensions, invasion depth, parametrial involvement, and nodal status often remain indeterminate. Moreover, substantial prognostic heterogeneity persists within the same FIGO stage classification, indicating the system’s insufficiency in comprehensively predicting clinical outcomes. These limitations necessitate the development of more sophisticated prognostic tools to enable precision medicine in LACC management.

Systemic inflammation critically regulates multiple oncogenic processes, including tumor initiation, progression, and metastasis.^6–7^ Hematological indicators such as white blood cell (WBC) count, neutrophil (Neu) count, lymphocyte (Lymph) count, and platelet (PLT) count reflect inflammatory status and have demonstrated prognostic value in oncology.^8^ However, the limited prognostic value of single inflammatory markers has prompted increasing adoption of composite biomarker analysis to improve predictive accuracy in clinical assessment.^9–11^ Tang et al.^12^ reported that the Systemic Immune-Inflammation Index (SII) outperformed the platelet to lymphocyte ratio (PLR) and WBC count in predicting CC survival, with the area under the receiver operating characteristic curve (AUC) reaching 0.728, 0.621, and 0.684, respectively. Similarly, the systemic inflammatory response index (SIRI) significantly outperformed PLR, neutrophil to lymphocyte ratio (NLR), and lymphocyte to monocyte ratio (LMR) in gallbladder cancer prognosis.^13^ In CC, systemic immune-inflammatory markers correlate strongly with lymph node metastasis, tumor invasion depth, and survival.^14–15^ Although the potential value of these indicators in CC has been preliminarily validated, the comprehensive predictive model based on immune-inflammatory markers still needs further refinement.

Nomograms provide an intuitive, quantitative method to predict clinical outcomes by integrating multiple prognostic variables via Cox regression analysis.^16^ These models align with precision medicine principles, offering superior accuracy, adaptability, and generalizability compared to traditional staging systems.^17–18^ To date, nomograms have been successfully applied across malignancies, including CC,^19–23^ and are increasingly adopted for survival prediction in both research and clinical practice.^24–26^

Machine learning (ML) has advanced the prognostic evaluation of CC. Parikh et al.^27^ developed ML models effectively identified high-risk cancer outpatients for short-term mortality, providing strong support for the integration of ML tools into clinical workflows. Ding et al.^28^ constructed a miRNA-based ML model stratifying CC patients by survival risk, while Wang et al.^29^ combined radiomics and ML to predict disease-free and OS post-CCRT in LACC. Similarly, Liu et al.^30^ demonstrated that the CT radiomics-clinical feature fusion models outperformed individual models in predicting progression-free survival. These results strongly indicate that the ML provides a powerful tool for the prognostic assessment of cervical cancer.

To evaluate the comparative performance of nomograms and ML models, we utilized a dynamic labeling method to assess survival outcomes in patients with LACC following radical radiotherapy. The dynamic labeling method affords a novel perspective based on the time axis, which helps in a more comprehensive assessment of the performance of different prognostic prediction methods. This approach not only captures the performance changes of predictive models at different time points but also quantifies the clinical significance of model improvements, providing a scientific basis for selecting the optimal predictive model. Through this systematic comparison, we aim to offer more precise and reliable guidance for the selection of tools in the prognostic assessment of patients with LACC following radical radiotherapy.

## Methods

### Study population

This study analyzed patients with LACC who underwent radical CCRT at the Radiotherapy Department of the Affiliated Cancer Hospital of Nanjing Medical University between September 2016 and December 2019. The inclusion criteria were as follows: (a) histopathological confirmation of cervical squamous cell carcinoma, adenocarcinoma, or adenosquamous carcinoma, staged IIB-IVA per the FIGO 2018 criteria^31^ (Patients originally staged by FIGO 2009 were reclassified per FIGO 2018 staging system); (b) absence of active inflammatory disease prior to treatment; (c) completion of radical CCRT, including external pelvic irradiation radiotherapy (EBRT), platinum-based concurrent chemotherapy, vaginal brachytherapy, and potential para-aortic EBRT, without previous surgery or radiotherapy; (d) absence of other malignancies, infections, hematologic conditions, or severe organ dysfunction; (e) complete blood tests conducted one week before treatment; and (f) age ≤ 80 years, a Karnofsky Performance Scale (KPS) score ≥ 70. Exclusion criteria comprised (a) patients who failed to complete the prescribed radiotherapy regimen and (b) patients with incomplete medical records. This study received ethical approval from the institutional review board of Jiangsu Cancer Hospital (No. 2023K-K087).

### Data collection

Clinicopathological parameters were systematically gathered, including basic patient information, smoking and obstetric histories, KPS score,^32^ FIGO 2018 staging, and pathological type. Hematological parameters included routine blood parameters and inflammation-related markers: WBC count, hemoglobin (HGB) level, red blood cell (RBC) count, PLT count, absolute and percentage neutrophil (Neu) count (N%), Lymph count, monocyte (Mono) count, and serum squamous cell carcinoma antigen (SCC-Ag). Additionally, we calculated several inflammation-related composite indices^33^: NLR, LMR, PLR, leukocyte to lymphocyte ratio (LLR), a combined index of PLT count and NLR (COP-NLR), SIRI, and SII. The COP-NLR scoring criteria are as follows: NLR < 3 and PLT < 300 × 10^9^/L is 0 points, NL >3 or PLT > 300 × 10^9^/L is 1 point, and NLR > 3 and PLT > 30 × 10^9^/L is 2 points. The SII and SIRI were calculated using established formulas.^34–35^

### Follow-up

Follow-up commenced one month after treatment completion, occurring every 3 months for the first 2 years, every 6 months from years 3 to 5, and annually thereafter until December 2024. OS, defined as the interval from pathological diagnosis to death or last contact, served as the primary endpoint.^36^

### Statistical analysis

Categorical variables were analyzed using the χ^2^ test or Fisher’s exact test (for expected frequencies <5). Continuous variables were compared using Student’s t-test for normal distribution and Mann–Whitney *U* test for non-normal distribution. Survival analysis employed the Kaplan–Meier method with log-rank testing for group comparisons. All analyses were performed using R (version 4.4.2), with statistical significance set at *P* < .05.

### Feature selection method

This study employed a multi-stage feature selection strategy. Spearman correlation analysis^37^ was initially performed to evaluate variable associations. Variables with significant correlation (absolute correlation coefficient > 0.8),^38^ were selectively included to prevent multicollinearity in the modeling process. Next, the Boruta algorithm^39–40^ was applied to select features significantly associated with clinical outcomes from the remaining variables. Finally, the features selected through the aforementioned steps were used to construct ML models and the nomogram.

### Nomogram development and validation

The proportional hazards assumption was verified using Schoenfeld residuals analysis, confirming the validity of the Cox regression model. A nomogram was then constructed to forecast the 3- and 5-year OS rates for patients with LACC. Internal validation through 1000 bootstrap resamples was performed to assess model robustness and prevent overfitting. The nomogram’s efficacy was assessed utilizing multiple performance metrics. The AUC was employed to gauge its discriminative capacity, where an AUC closer to 1 signifies superior discriminative performance. Calibration curves evaluated the agreement between predicted and observed outcomes, with optimal alignment indicated by congruence with the reference diagonal. Decision curve analysis (DCA)^41^ was utilized to ascertain the net clinical benefit of the nomogram and to comprehensively evaluate its clinical predictive value.

### Risk stratification ability of the model

We employed a risk stratification strategy that integrates nomogram risk scores with recursive partitioning, combined with survival analysis to validate the effectiveness of prognostic grouping. First, we extracted the linear predictor values from the nomogram as individualized risk scores. Subsequently, the conditional inference tree algorithm was used to recursively partition the risk scores, determining the optimal risk cutoff by maximizing survival differences. Under the constraints of a minimum node sample size (≥30 cases) and a maximum partition depth (2 splits), the algorithm automatically identified cutoff points that maximized the log-rank test statistic between groups, ensuring clinical interpretability and generating 3 categories: low, medium, and high risk. When automatic partitioning failed to identify effective cutoffs, the tertiles of the risk scores were used as a robust alternative. Using the predetermined optimal cutoff values, patients in the training cohort were categorized into low-, medium-, and high-risk groups. Kaplan–Meier analysis with log-rank testing evaluated survival differences among these groups.

### Development and evaluation of ML models

Patient survival status at final follow-up was utilized as the primary classification outcome. We developed predictive models based on 5 ML models: Logistic Regression, Random Forest, Extreme Gradient Boosting (XGBoost), Decision Tree, and Light Gradient Boosting Machine (LightGBM).

During model development, the dataset was randomly split into training (70%) and validation (30%) cohorts. Hyperparameter optimization was performed through 10-fold cross-validation on the training set, while model performance was assessed on the independent validation set using 5 established metrics for binary classification: accuracy, AUC, precision, recall, and F1-score. These metrics were calculated from the confusion matrix components: true positives (TP), true negatives (TN), false positives (FP), and false negatives (FN). Accuracy quantified overall correct predictions (TP+TN) across all cases. The AUC, representing model discriminative capacity, was calculated by plotting sensitivity against 1-specificity across all classification thresholds, with higher values indicating superior positive-negative differentiation. Recall measures the proportion of correctly identified non-survivors, whereas specificity reflects the accuracy of survivor identification. Higher precision indicates that the model produces more TP results than FP results, providing more reliable predictions. The F1-score represents the harmonic balance between precision and recall.


(1) $$\mathrm{Accuracy}=\frac{\mathrm{TP}+\mathrm{TN}}{\mathrm{TP}+\mathrm{TN}+\mathrm{FP}+\mathrm{FN}}$$

(2) $$\mathrm{Precision}=\frac{\mathrm{TP}}{\mathrm{TP}+\mathrm{FP}}$$

(3) $$\mathrm{Recall}=\frac{\mathrm{TP}}{\mathrm{TP}+\mathrm{FN}}$$

(4) $$F1‐\mathrm{Score}=\frac{2\times \mathrm{Precision}\times \mathrm{Recall}}{\mathrm{Precision}+\mathrm{Recall}+\mathrm{FP}}$$

### Comparative analysis of nomogram and ML models

This study compared the nomogram’s predictive performance with the ML model using dynamic labeling method,^42^^,^^56^ wherein survival status was continuously updated for all validation cohort patients at every follow-up interval. Patients who died before the specified time point were labeled as “deceased,” while those who survived beyond that time point were labeled as “alive.” For instance, at the 5-year follow-up, “deceased” was defined as death within 5 years of initial treatment, and “alive” as survival for at least 5 years. Patients lost to follow-up prior to a specific time point were excluded from the validation set. Based on the relabeled validation set, we evaluated the model’s accuracy using Formula (1) and developed a time-dependent accuracy curve to evaluate the predictive performance of the nomogram against the ML model. Among 5 models, the ML model with the highest accuracy (according to Formula 1) was selected for comparative analysis with the nomogram.

## Results

### Patient characterization

The study enrolled 300 patients with LACC, comprising 74 patients (24.67%) with stage IIB, 99 patients (33%) with stage IIIB, 116 patients (38.67%) with stage IIIC1, and 11 patients (3.67%) with stage IIIC2. Median diagnosis age was 59.5 years (range: 55-66 years). Complete baseline characteristics are presented in Table S1.

### Selection of key features

Spearman rank correlation coefficients were assessed for 26 survival-associated variables (Figure S1). The results indicated high correlations (absolute *r* > 0.8) between certain variables, such as WBC and Neu (*r* = 0.90), N% and NLR (*r* = 0.86), N% and LLR (*r* = 0.81), SIRI and LMR (*r* = 0.84), SII and NLR (*r* = 0.86), SII and LLR (*r* = 0.85), and NLR and LLR (*r* = 0.97). To avoid multicollinearity issues, Neu, NLR, LLR, and LMR were removed following discussions among more than 2 experts.

The Boruta method was applied to an additional 22 variables, identifying 5 variables—WBC, SII, N%, SIRI, and PLR—as important (Figure S2). Given the clinical significance and widespread recognition of the FIGO staging system, the research team incorporated it into the nomogram and ML models. Ultimately, these selected features were then employed to construct the predictive tools.

Before employing the Cox regression model, the assumption of proportional hazards was validated through Schoenfeld residual plots (Figure S3). The results demonstrated that the Schoenfeld residuals were evenly distributed and independent of survival time, with the overall model’s *P*-value being .33 and each variable’s *P*-value exceeding .05. This indicates that these 6 variables satisfy the proportional hazards assumption, meaning their impact on survival remains constant over time, thus supporting the use of the Cox regression model.

### Analysis and validation of the nomogram

**Figure 1.**
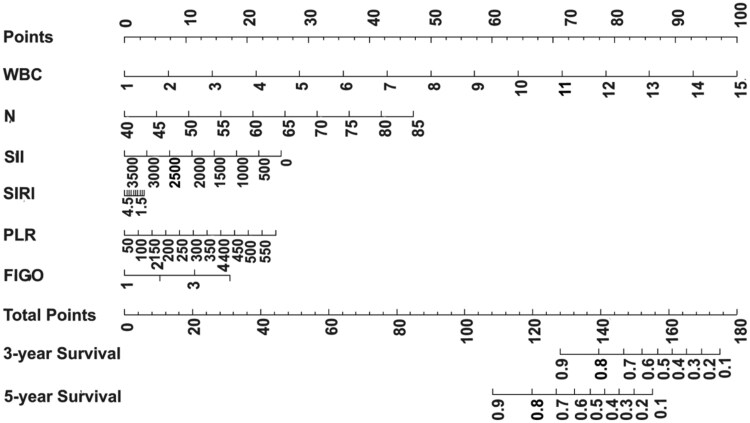
Nomogram for predicting 3- and 5-year overall survival.

**Figure 2.**
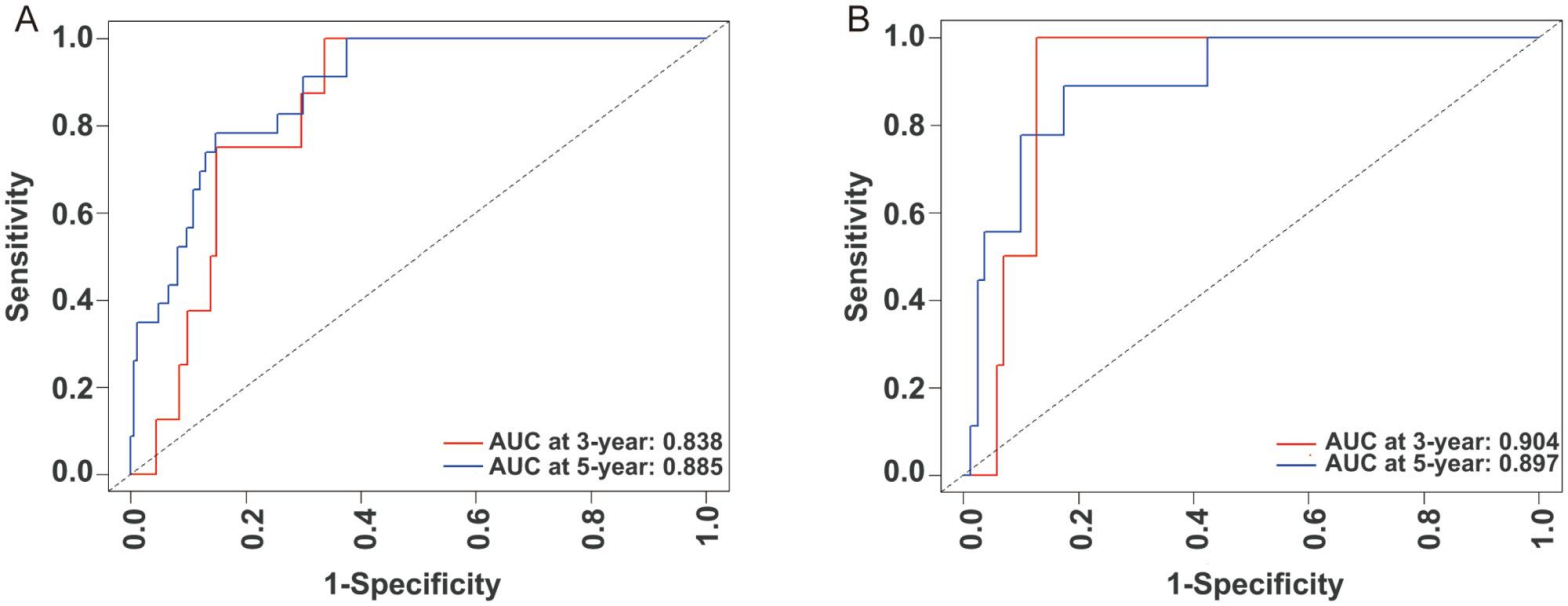
ROC curves of the nomogram for predicting 3- and 5-year OS. (A) The training cohort. (B) The validation cohort.

**Figure 3.**
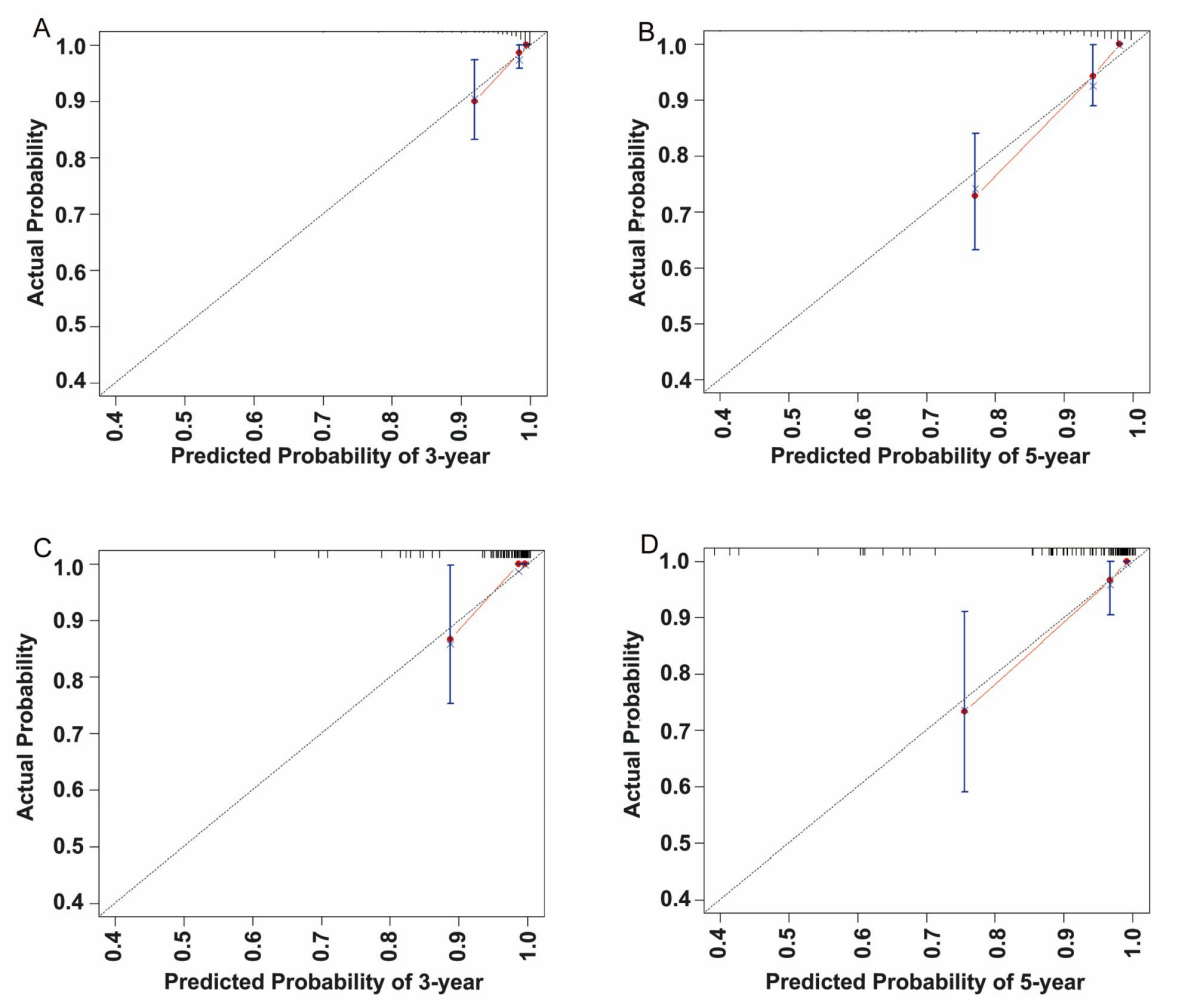
Calibration curves for 3- and 5-year OS. (A) Calibration curve for 3-year OS in the training cohort. (B) Calibration curve for 5-year OS in the training cohort. (C) Calibration curve for 3-year OS in the validation cohort. (D) Calibration curve for 5-year OS in the validation cohort.

**Figure 4.**
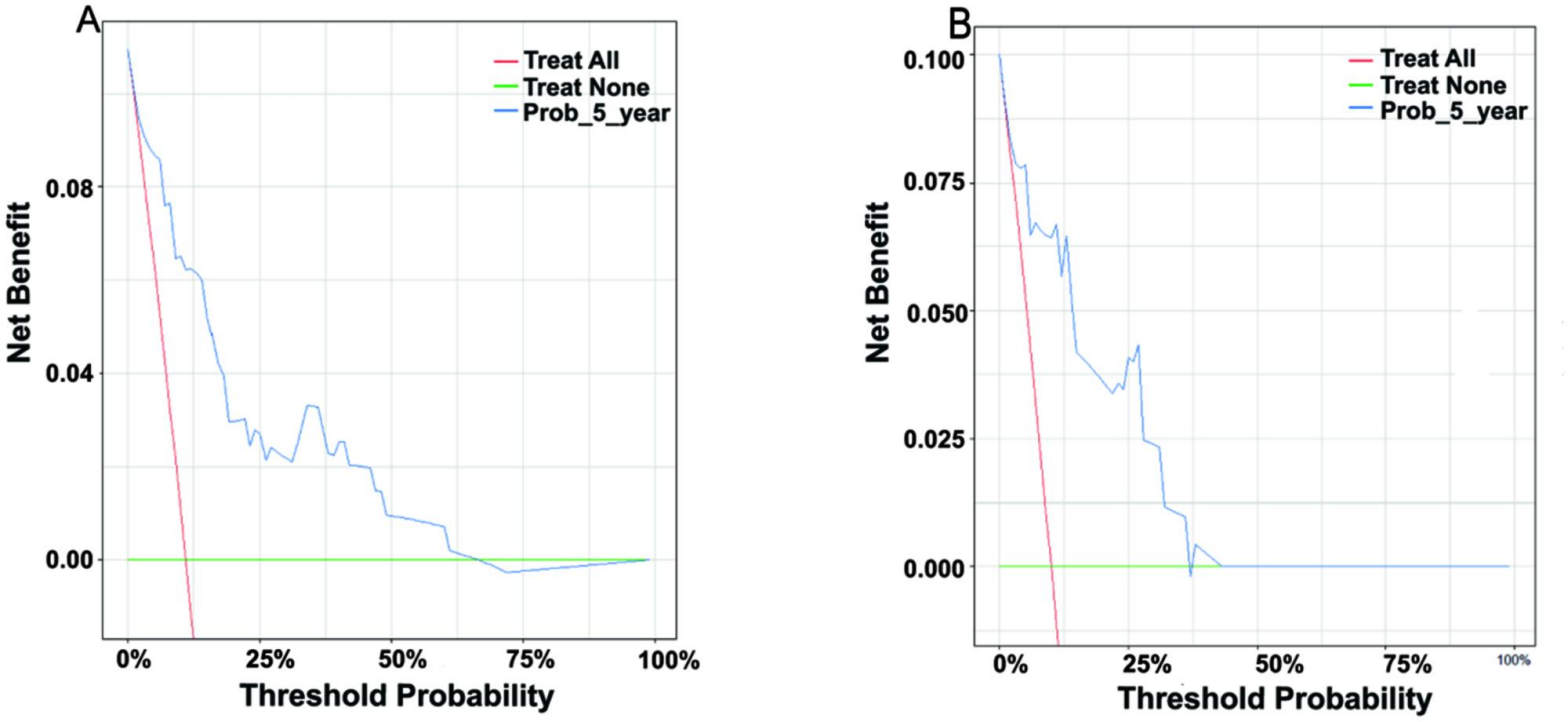
Decision curve analysis of the nomogram predicting 5-year OS. (A) Training cohort. (B) Validation cohort.

A nomogram was developed to estimate the 3- and 5-year OS rates for patients in the training set (Figure 1), with variables weighted according to their prognostic significance. The cumulative score, derived from summing all individual points, corresponds to specific survival probabilities projected on the nomogram’s probability axis. The nomogram’s performance was internally validated. Figure 2A and 2B present the ROC curves, with the red and blue curves representing the predictions for 3- and 5-year OS, respectively. The nomogram demonstrated AUC values of 0.838 (3-year) and 0.885 (5-year) in the training set, and 0.904 (3-year) and 0.897 (5-year) in the validation set. These results indicate that the nomogram’s high accuracy for OS, with calibration curves showing excellent agreement between predicted and observed 3- and 5-year OS rates in both cohorts (Figure 3A-D). Moreover, the DCA for the 5-year survival prediction revealed that the nomogram provides a clinical net benefit within a probability threshold range of 0 to 0.66 in the training set and 0 to 0.38 in the validation set. Notably, within the commonly accepted clinical decision threshold range of 0.25-0.40,^43^ the nomogram consistently conferred stable clinical net benefits in both sets, thereby serving as an ideal aid for treatment decision-making (Figure 4).

### Risk stratification capability of the nomogram

**Figure 5.**
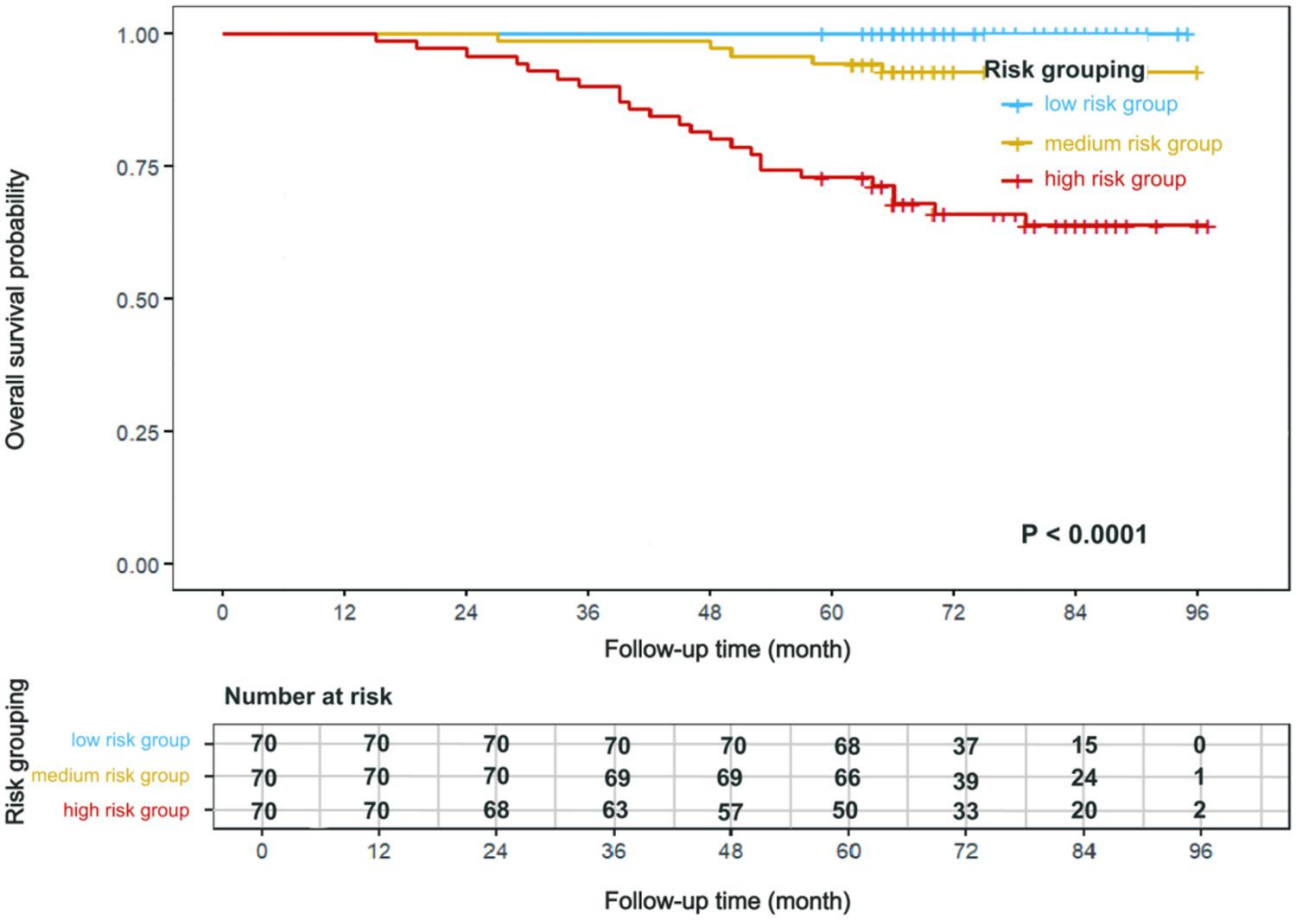
Comparison of survival curves for 3 risk groups generated by the nomogram.

We extracted the linear predictor values from the nomogram as individualized risk scores. These risk scores were then subjected to recursive partitioning using the conditional inference tree algorithm, which identified 2 optimal cut-off points by maximizing survival differences (low/medium risk cut-off: −0.6; medium/high risk cut-off: 0.5). Patients in the training set were stratified into low-, medium-, and high-risk groups. Kaplan–Meier survival curves revealed significant differences in survival rates among the groups (log-rank χ^2^ = 40.31, *P* < .0001), effectively identifying those with poor prognosis (Figure 5).

### Evaluation of ML model

The performance metrics of the 5 ML models are summarized in Table 1. The logistic regression model emerged as the optimal model, offering the best balance of accuracy (0.933), discriminative power (AUC = 0.981), and clinical utility (recall = 0.882, F1-score = 0.938). Its near-perfect recall renders it particularly suitable for clinical scenarios with low tolerance for missed diagnoses, such as early cancer screening. The high F1-score, which harmonizes both precision and recall, indicates that the model achieves this high sensitivity without an untenable number of false positives, making it both a sensitive and reliable tool. The model’s marginally lower precision, a deliberate trade-off for high recall, may result in a manageable rate of false positives, which is often an acceptable compromise in critical screening contexts.

**Table 1.** Cross-validation performance metrics of machine-learning models in the validation group.

Model	Accuracy	AUC	Precision	Precision	F1-score
**Logistic regression**	0.933	0.981	0.882	1.000	0.938
**Random forest**	0.900	0.976	0.909	0.889	0.899
**XGBoost**	0.856	0.956	0.900	0.800	0.847
**Decision tree**	0.844	0.868	0.943	0.733	0.825
**LightGBM**	0.900	0.963	0.929	0.867	0.897

Abbreviations: AUC, area under the curve; LightGBM, light gradient boosting machine.

### Comparison of nomogram and logistic regression model

**Figure 6.**
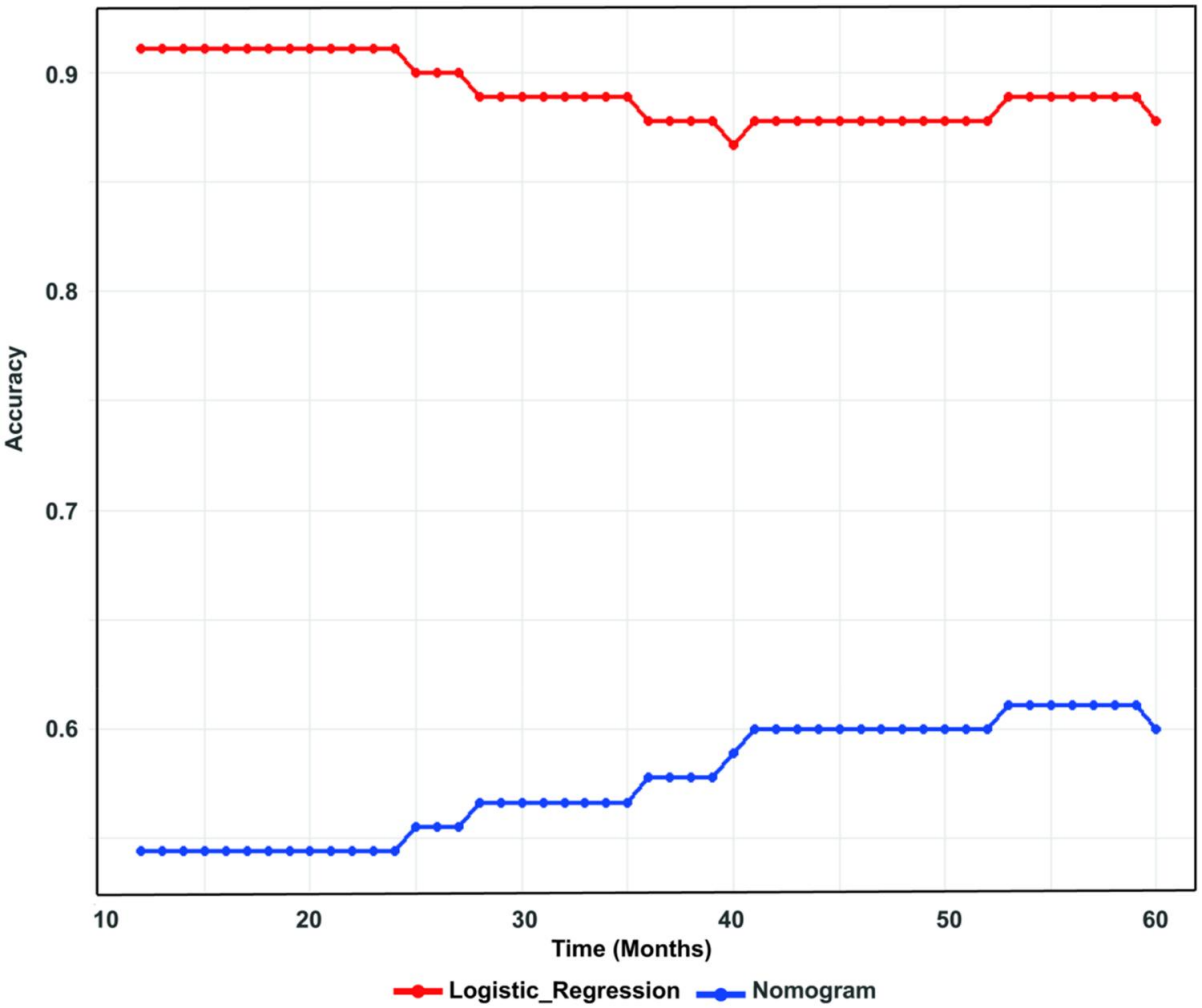
Time-dependent accuracy of the nomogram and logistic regression model.

During the follow-up period from months 12 to 60, we re-labeled the survival status of the validation set and assessed the predictive accuracy (Formula 1), subsequently deriving time-dependent accuracy metrics (Table S2 and Figure 6). The nomogram’s predictive accuracy increased from 12 to 60 months, reaching a peak of 0.611 between months 53 and 59. Conversely, the logistic regression model’s accuracy decreased during this period, with its highest accuracy of 0.911 observed between months 12 and 24. Throughout the follow-up period, the logistic regression model consistently demonstrated superior predictive accuracy compared to the nomogram.

## Discussion

CC as the world’s fourth most prevalent female malignancy, accounted for 661,000 new cases and 348,000 deaths in 2022. Its incidence and mortality rates are showing a continuous upward trend in China.^1^^,^^44^ For LACC, despite the use of the CCRT regimen recommended by the National Comprehensive Cancer Network guidelines, nearly one-third of patients still experience recurrence within 5 years,^45–46^ posing significant threating for females health globally. This underscores the urgent need for non-invasive and easily accessible prognostic models to optimize treatment selection and improve clinical outcomes. Emerging evidence demonstrates that systemic immune-inflammatory markers critically influence tumor microenvironments by facilitating proliferation, angiogenesis, and metastasis while concurrently suppressing adaptive immune responses.^47–49^ Despite established prognostic significance across multiple cancers,^50–52^ their predictive utility in LACC remains understudied. Based on this, we integrated immune-inflammatory markers with the clinical staging system to construct the nomogram and ML models, aiming to optimize the prognostic assessment for LACC after radical radiotherapy.

Feature selection employed Spearman’s rank correlation coefficient to reduce multicollinearity, followed by the Boruta algorithm to identify variables significantly associated with OS. Ultimately, 5 variables—WBC, SII, N%, SIRI, and PLR—were confirmed as important and were included, along with the FIGO staging system, in the construction of the nomogram and ML models. The Cox regression-based nomogram showed good discrimination (AUC >0.8) and calibration, though DCA revealed limited clinical net benefit for high-risk thresholds. This limitation may stem from (1) high-risk patients often exhibit complex molecular heterogeneity, which is challenging to capture with inflammation markers alone; (2) the limited sample size of the high-risk cohort, which diminishes confidence in the model’s predictions for this group; and (3) high-risk patients may involve mechanisms of treatment resistance, and the current model does not incorporate related molecular biomarkers.

We employed a recursive partitioning algorithm to determine the critical values for risk stratification, successfully segregating the patients into low-, medium-, and high-risk cohorts with significant survival differences. The Kaplan–Meier survival analysis confirmed a substantial survival gradient across the groups, indicating the nomogram’s efficacy in identifying patients at risk of unfavorable outcomes. This method has 2 major advantages over the traditional X-tile software^53^: (1) it uses adaptive partitioning based on survival time and event information, avoiding the bias of subjective threshold setting; (2) it automatically optimizes the grouping strategy by maximizing information gain, enhancing the clinical prognostic differentiation efficacy; and (3) it supports multi-stage risk stratification, which is more aligned with actual clinical needs.

The logistic regression model demonstrated optimal predictive performance, achieving an accuracy of 0.933, AUC of 0.981, recall of 1.000, and F1-score of 0.938. This combination of strong discriminatory capacity and minimal overfitting is particularly valuable for medical diagnostic applications where reliable positive case detection is essential. In contrast, the decision tree underperformed, presenting the lowest accuracy (0.844), AUC (0.868), and F1-score (0.825). Despite its high precision (0.943), a low recall rate (0.733) signified predictive bias, hinting at a conservative bias possibly due to over-pruning or limited depth, risking the overlook of positive cases. Accuracy in predictive models is critical for clinical application.^54–55^ Therefore, we further compared the ML model with the highest accuracy to the nomogram. Dynamic time validation confirmed logistic regression’s superiority over the nomogram, particularly in early-phase (12-24 months) risk prediction (AUC = 0.911), enabling timely intervention.

Our findings align with Alabi et al.’s^56^ observations in their tongue cancer research. However, their research only evaluated model performance at a single time point, whereas our study employed a dynamic labeling method to assess performance at multiple time points, providing a more comprehensive comparison. Currently, many studies suggest that ML models outperform traditional methods in predicting adverse events in patients. Pezel et al.^57^ demonstrated that a ML model combining multimodal data outperformed traditional methods in predicting adverse events in coronary artery disease. However, recent studies have also pointed out that ML models do not necessarily outperform traditional models,^58^ especially in low-dimensional data scenarios, where traditional models may achieve comparable performance. These findings suggest that while ML excels in certain scenarios, its clinical application should be approached with caution, particularly in scenarios with limited data or high model complexity.

Several limitations should be noted. Firstly, the single-center retrospective design introduces potential selection bias, and the modest sample size may limit generalizability. Secondly, reliance on internal bootstrapping validation without external cohort verification could potentially overestimate the model’s efficacy. Additionally, the study lacks mechanistic validation of the observed associations between inflammatory markers and prognosis through pathway analysis or experimental approaches. Future research should ideally involve multi-center, prospective trials to increase sample size and improve generalizability. Incorporating external validation cohorts to confirm the model’s robustness and clinical relevance. Additionally, the investigation of advanced ML algorithms, including deep learning, and integrating multimodal data (such as genomics, imaging, etc.) could further improve predictive accuracy.

## Conclusions

This study validated the effectiveness of a prognostic model constructed using immune-inflammatory markers combined with the FIGO staging system for predicting survival in LACC patients after radical radiotherapy. The nomogram demonstrated acceptable discriminative capacity and predictive accuracy in the overall evaluation, providing a visual tool for clinical prognostic assessment. Dynamic time analysis revealed that the logistic regression model outperformed the nomogram at all time points from 12 to 60 months, potentially offering a more precise and reliable tool for guiding prognostic assessment in patients with LACC and contributing to the optimization of clinical decision support systems.

## Supplementary Material

oyag139_Supplementary_Data

## Data Availability

The data underpinning this study, consisting of in-hospital patient records, are intended for research purposes and are not available to the public.
