# Supplementary material for: Prognostic utility of systemic immune-inflammation markers in locally advanced cervical cancer undergoing radical radiotherapy
Source: Oncologist. 2026 Apr 21;31(5):oyag139. doi: 10.1093/oncolo/oyag139 (PMC13131946; doi:10.1093/oncolo/oyag139)
Supplement: oyag139_Supplementary_Data [file oyag139_supplementary_data.zip › Table S3.docx]

**Supplemental Table S3**: Glossary

| **Full Name** | **Abbreviation** |
| --- | --- |
| Locally advanced cervical cancer | LACC |
| Machine learning | ML |
| Decision curve analysis | DCA |
| Systemic Immune-Inflammation Index | SII |
| Systemic Inflammatory Response Index | SIRI |
| Platelet to Lymphocyte Ratio | PLR |
| Neutrophil to Lymphocyte Ratio | NLR |
| Lymphocyte to Monocyte Ratio | LMR |
| Extreme Gradient Boosting | XGBoost |
| Light Gradient Boosting Machine | LightGBM |
