# Supplementary material for: Prognostic utility of systemic immune-inflammation markers in locally advanced cervical cancer undergoing radical radiotherapy
Source: Oncologist. 2026 Apr 21;31(5):oyag139. doi: 10.1093/oncolo/oyag139 (PMC13131946; doi:10.1093/oncolo/oyag139)
Supplement: oyag139_Supplementary_Data [file oyag139_supplementary_data.zip › TableS1.docx]

**Supplemental Table S1**: Characteristics of the training and validation groups

| **Variable** | **Training Group(210)** | **Validation Group(90)** | **P-value** |
| --- | --- | --- | --- |
| **Age (years)** | 60（55，66） | 59（55，65.75） | 0.993 |
| **Smoking, n (%)** |  |  | 0.809 |
| No | 188（89.52） | 79（87.78） |  |
| Yes | 22（10.48） | 11（12.22） |  |
| **BMI (kg/m^2^)** | 25（2，27） | 25（22，27） | 0.887 |
| **P** |  |  | 0.059 |
| ≤ 3 | 194（92.38） | 76（84.44） |  |
| ＞3 | 16（7.62） | 14（15.56） |  |
| **G** |  |  | 0.307 |
| ≤ 3 | 127（60.48） | 48（53.33） |  |
| ＞3 | 83（39.52） | 42（46.67） |  |
| **FIGO** |  |  | 0.957 |
| IIB | 52（24.76） | 22（24.44） |  |
| IIIB | 71（33.81） | 28（31.11） |  |
| IIIC1 | 79（37.62） | 37（41.11） |  |
| IIIC2 | 8（3.81） | 3（3.33） |  |
| **Histology** |  |  | 1 |
| Adeno/Others | 7（3.33） | 3（3.33） |  |
| SCC | 203（96.67） | 87（96.67） |  |
| **KPS, n(%)** |  |  | 1 |
| ＜80 | 21（10） | 9（10） |  |
| ≥ 80 | 189（90） | 81（90） |  |
| **HPV Infection,n (%)** |  |  | 0.485 |
| No | 39（18.57） | 13（14.44） |  |
| Yes | 171（81.43） | 77（85.56） |  |
| **WBC (10⁹/L)** | 6.28（5.16，7.1） | 6.13（5.14，7.34） | 0.819 |
| **HGB (g/L)** | 123（110，132） | 118.5（107.25，130） | 0.141 |
| **RBC (10^12^/L)** | 4.28（3.93，4.55） | 4.22（3.81，4.5） | 0.195 |
| **PLT (10⁹/L)** | 255（212.25，309.25） | 266（206.25，301.75） | 0.837 |
| **Neu (10⁹/L)** | 3.94（3.2，4.98） | 3.99（3.13，5.03） | 0.908 |
| **N%** | 65.92（59.44，71.93） | 65.99（58.41，72.11） | 0.897 |
| **Lymph (10⁹/L)** | 1.6（1.19，1.94） | 1.48（1.21，1.92） | 0.618 |
| **Mono (10⁹/L)** | 0.34（0.25，0.44） | 0.34（0.27，0.5） | 0.231 |
| **SCC-Ag (μg/L)** | 6.35（2.73，16.55） | 6.8（2.92，21.37） | 0.329 |
| **SIRI** | 0.92（0.6，1.33） | 0.93（0.65，1.47） | 0.392 |
| **SII** | 668.41（457.1，997.38） | 648.95（455.15，890.07） | 0.807 |
| **NLR** | 2.57（1.86，3.73） | 2.62（1.84，3.73） | 0.850 |
| **PLR** | 163.15（124.44，219.29） | 175.83（128.49，224.56） | 0.870 |
| **LMR** | 0.22（0.17，0.3） | 0.23（0.18，0.32） | 0.333 |
| **LLR** | 3.93（3.14，5.25） | 4.01（3.11，5.02） | 0.913 |
| **COP-NLR, n (%)** |  |  | 0.527 |
| 000 | 97（46.19） | 44（48.89） |  |
| 001 | 92（43.81） | 34（37.78） |  |
| 002 | 21（10） | 12（13.33） |  |
| **Maximum Tumor Diameter (cm)** | 5.2±1.02 | 5.39±0.99 | 0.137 |
| **Death, n (%)** |  |  | 1 |
|  | 181（86.19） | 78（86.67） |  |
|  | 29（13.81） | 12（13.33） |  |

Abbreviations: Parity, P; Gravidity, G; KPS, Karnofsky Performance Scale; SCC, Squamous Cell Carcinoma; Adeno, Adenocarcinoma
