# Supplementary material for: Prognostic utility of systemic immune-inflammation markers in locally advanced cervical cancer undergoing radical radiotherapy
Source: Oncologist. 2026 Apr 21;31(5):oyag139. doi: 10.1093/oncolo/oyag139 (PMC13131946; doi:10.1093/oncolo/oyag139)
Supplement: oyag139_Supplementary_Data [file oyag139_supplementary_data.zip › TableS2.docx]

**Table S2**. Time-dependent Accuracy of the Nomogram and Logistic Regression Model Within 1-5 Years.

| **Follow-up time**  **(month)** | **Accuracy** | |
| --- | --- | --- |
|  | **Nomogram** | **Logistic Regression** |
| 12 | 0.544 | 0.911 |
| 13 | 0.544 | 0.911 |
| 14 | 0.544 | 0.911 |
| 15 | 0.544 | 0.911 |
| 16 | 0.544 | 0.911 |
| 17 | 0.544 | 0.911 |
| 18 | 0.544 | 0.911 |
| 19 | 0.544 | 0.911 |
| 20 | 0.544 | 0.911 |
| 21 | 0.544 | 0.911 |
| 22 | 0.544 | 0.911 |
| 23 | 0.544 | 0.911 |
| 24 | 0.544 | 0.911 |
| 25 | 0.556 | 0.900 |
| 26 | 0.556 | 0.900 |
| 27 | 0.556 | 0.900 |
| 28 | 0.567 | 0.889 |
| 29 | 0.567 | 0.889 |
| 30 | 0.567 | 0.889 |
| 31 | 0.567 | 0.889 |
| 32 | 0.567 | 0.889 |
| 33 | 0.567 | 0.889 |
| 34 | 0.567 | 0.889 |
| 35 | 0.567 | 0.889 |
| 36 | 0.578 | 0.878 |
| 37 | 0.578 | 0.878 |
| 38 | 0.578 | 0.878 |
| 39 | 0.578 | 0.878 |
| 40 | 0.589 | 0.867 |
| 41 | 0.600 | 0.878 |
| 42 | 0.600 | 0.878 |
| 43 | 0.600 | 0.878 |
| 44 | 0.600 | 0.878 |
| 45 | 0.600 | 0.878 |
| 46 | 0.600 | 0.878 |
| 47 | 0.600 | 0.878 |
| 48 | 0.600 | 0.878 |
| 49 | 0.600 | 0.878 |
| 50 | 0.600 | 0.878 |
| 51 | 0.600 | 0.878 |
| 52 | 0.600 | 0.878 |
| 53 | 0.611 | 0.889 |
| 54 | 0.611 | 0.889 |
| 55 | 0.611 | 0.889 |
| 56 | 0.611 | 0.889 |
| 57 | 0.611 | 0.889 |
| 58 | 0.611 | 0.889 |
| 59 | 0.611 | 0.889 |
| 60 | 0.600 | 0.878 |
