# Supplementary material for: Prognostic utility of systemic immune-inflammation markers in locally advanced cervical cancer undergoing radical radiotherapy
Source: Oncologist. 2026 Apr 21;31(5):oyag139. doi: 10.1093/oncolo/oyag139 (PMC13131946; doi:10.1093/oncolo/oyag139)
Supplement: oyag139_Supplementary_Data [file oyag139_supplementary_data.zip › Figure S1-3.docx]

**Supplemental Figure S1.** Spearman Correlation Heatmap of Variables

**Supplemental Figure S2.** Variable Selection Using Boruta Method


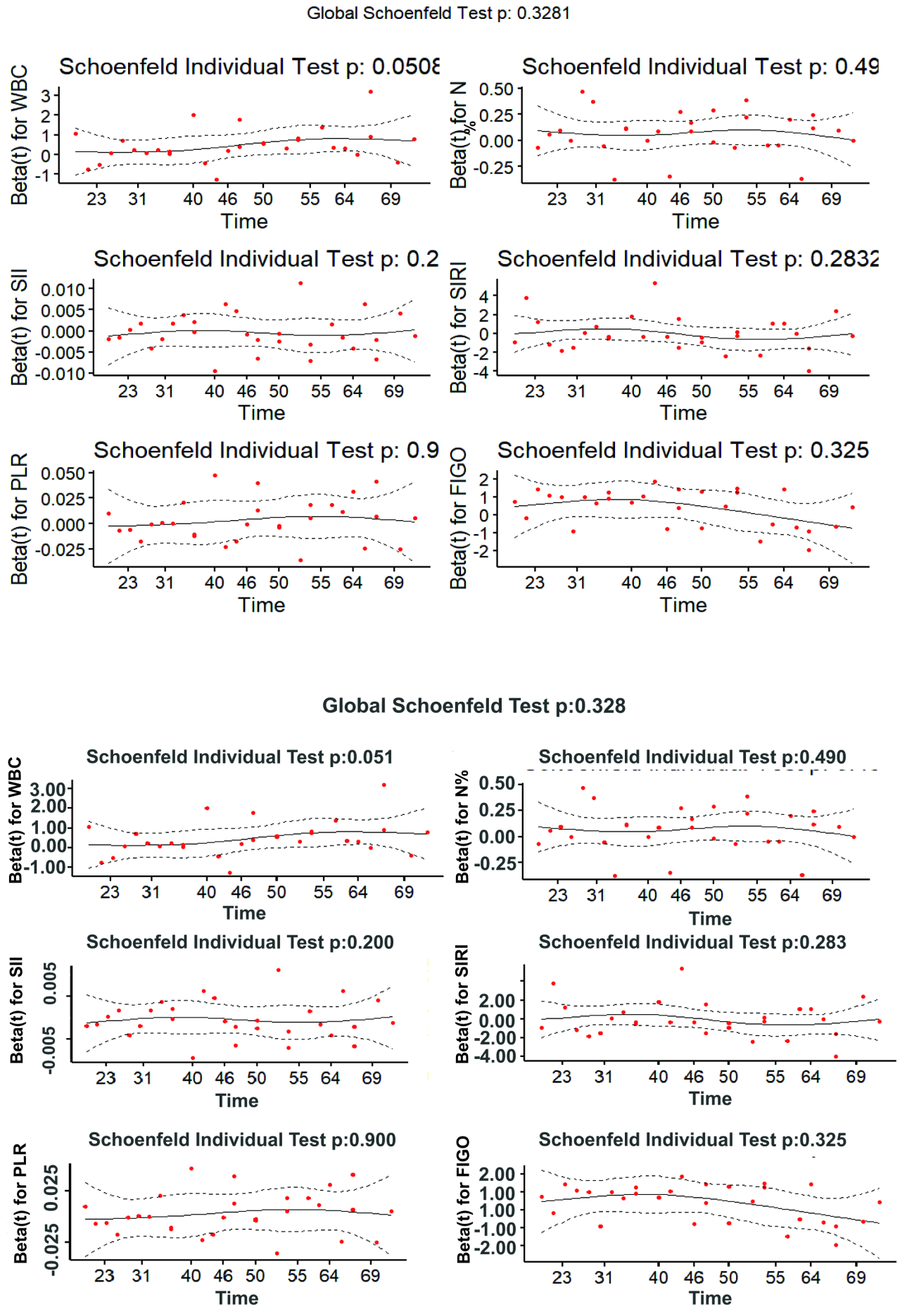


**Supplemental Figure S3.** Schoenfeld Residuals Test for Proportional Hazards Assumption
